# Reentrant processing mediates object substitution masking: comment on Põder (2013)

**DOI:** 10.3389/fpsyg.2014.00819

**Published:** 2014-08-04

**Authors:** Vincent Di Lollo

**Affiliations:** Department of Psychology, Simon Fraser University, Burnaby, BCCanada

**Keywords:** visual masking, object substitution masking, feed-forward, reentrant processing, attention

## Abstract

Object-substitution masking (OSM) occurs when a target stimulus and a surrounding mask are displayed briefly together, and the display then continues with the mask alone. Target identification is accurate when the stimuli co-terminate but is progressively impaired as the duration of the trailing mask is increased. In reentrant accounts, OSM is said to arise from iterative exchanges between brain regions connected by two-way pathways. In an alternative account, OSM is explained on the basis of exclusively feed-forward processes, without recourse to reentry. Here I show that the feed-forward account runs afoul of the extant phenomenological, behavioral, brain-imaging, and electrophysiological evidence. Further, the feed-forward assumption that masking occurs when attention finds a degraded target is shown to be entirely *ad hoc*. In contrast, the evidence is uniformly consistent with a reentrant-processing account of OSM.

*Visual masking* refers to an impairment in the perception of a briefly presented object (the target) by the presentation of a second object (the mask) in close spatiotemporal proximity. The present work is concerned with a form of masking known as *object-substitution masking* (OSM) that occurs when a brief simultaneous display of the target and the mask continues with a display of the mask alone (Di Lollo et al., 2000).

**Figure 1** illustrates the basic OSM paradigm. The display sequence begins with a brief presentation of a variable number of rings, each with a gap in one of the four cardinal orientations. Observers indicate the orientation of the gap in the target ring, which is singled out by four surrounding small dots that act as both cue and mask. After a brief exposure, all elements in the display are turned off except for the four dosts which remain on view for a variable period of up to several hundred ms. When the target and the mask terminate together (i.e., when there is no trailing display of the four dots alone) the target is identified accurately. Masking develops rapidly, however, as the duration of the trailing four-dot mask is increased up to about 200 ms (see **Figure 2**).

**FIGURE 1:**
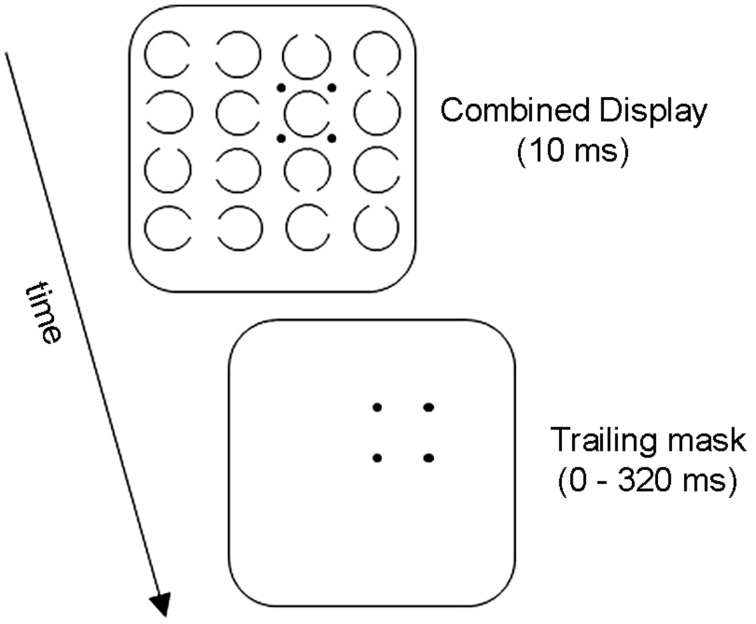
**Display sequence in a conventional OSM paradigm**.

**FIGURE 2:**
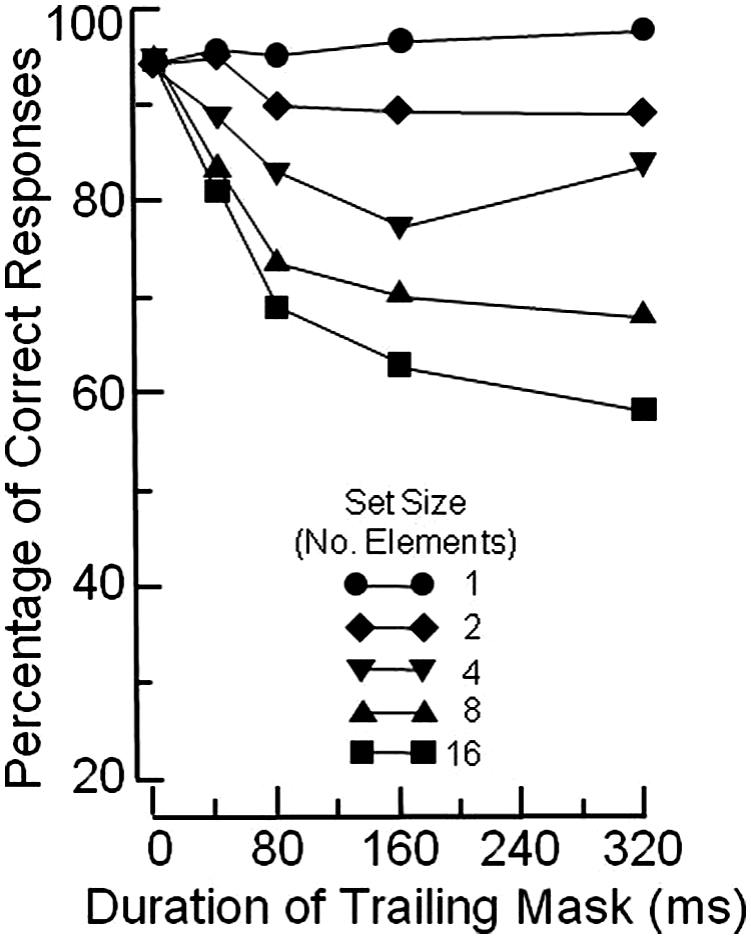
**FIGURE 2. Mean percentage of correct identifications of the orientation of the gap in the target ring, using the paradigm illustrated in **Figure 1****. Redrawn from Figure 4 in Di Lollo et al. (2000).

Early theoretical accounts of OSM were couched in terms of reentrant processes that take place after an initial feed-forward sweep (Di Lollo et al., 2000; Lleras and Moore, 2003). More recently, an exclusively feed-forward account has been proposed by Põder (2013). That account is examined and questioned in the present work.

## A REENTRANT ACCOUNT OF OSM

In the conventional OSM paradigm (see **Figure 1**) the target and the mask have a common onset; therefore, no unique onset transient is generated by the mask. This rules out onset transients as a source of masking (e.g., Breitmeyer and Ganz, 1976; see Di Lollo et al., 2000, for a more detailed account of the role of transient responses in OSM). Rather, OSM is thought to be mediated by reentrant signals between brain regions connected by two-way pathways.

In the feed-forward sweep, the neural activity triggered by the initial display ascends to higher brain regions, where it activates a large number of perceptual hypotheses that are in some way compatible with the sensory input. The perceptual hypotheses then descend to lower levels, where they attempt to match themselves to the pattern of ongoing activity through a process of correlation. Matches that yield low correlations are discarded, whereas the hypothesis that yields the highest correlation is confirmed and eventually leads to conscious awareness (Mumford, 1991, 1992; Grossberg, 1995; Di Lollo et al., 2000).

Masking occurs when a mismatch arises between the reentrant signals and the ongoing activity at the lower level. At short durations of the trailing mask, the reentrant signals find a pattern of ongoing low-level activity that, although decayed, is of relatively uniform strength. Notably, the brief additional display of the four dots causes the low-level representation of the mask to be only slightly stronger than that of the target. In this case, little or no masking occurs because the similarity between the reentrant hypothesis and the low-level representation allows for an adequate correlation. This leads to confirmation of that perceptual hypothesis, and to relatively accurate target identification, as illustrated by the short-mask-duration points in **Figure 2**.

In contrast, at long durations of the trailing mask, the reentrant signals find a pattern of ongoing low-level activity of non-uniform strength. To wit, the representation of the target has decayed, but the mask remains at full strength because of the continued external input. This mismatch reduces the correlation with the reentrant hypothesis, which consists of a representation of the target and the mask at uniform strength. The ensuing low correlation causes the current perceptual hypothesis to be discarded, and a new “mask-alone” hypothesis to be generated, with consequent impairment of target identification, as illustrated by the long-duration points in **Figure 2**.

## PÕDER’S FEED-FORWARD ACCOUNT OF OSM

A simpler, strictly feed-forward account has been proposed by Põder (2013). The account is based on two assumptions. First, the continued presence of the mask after the offset of the initial display is held to add noise at the target’s internal representation, causing its signal-to-noise (S/N) ratio to be reduced. Because of temporal integration, the noise continues to grow while the mask remains on view. For this reason, the reduction in S/N ratio is said to be proportional to the exposure duration of the trailing mask. Second, masking is assumed to occur when attention is deployed to the target location. Upon its deployment, attention finds a degraded representation of the target due to reduced S/N ratio, and accuracy of target identification is impaired correspondingly.

The two assumptions were embodied in a computational model (Põder, 2013) that provided an excellent fit for the OSM data reported by Di Lollo et al. (2000; see present **Figure 2**). This buttressed the claim that OSM can be explained in strictly feed-forward terms, without recourse to reentry.

Põder’s (2013) assumptions are examined in the remainder of this article. The assumption that the trailing mask reduces the target’s S/N ratio is shown to run afoul of the phenomenological, behavioral, brain-imaging, and electrophysiological evidence. Further, the assumption that masking occurs when attention finds a degraded target is shown to be *ad hoc*.

## ASSUMPTION OF REDUCED TARGET S/N RATIO

Põder’s (2013) account of how the noise generated by the extended presentation of the four-dot mask may affect the target’s internal representation does not draw a distinction between sensory noise and non-specific internal “system” noise. In what follows, I endeavor to show that externally generated noise stemming from the prolonged exposure of the four-dot-mask is inadequate as a determinant of OSM. Furthermore, an account based on non-specific internally generated noise is just as inadequate^1^.

Non-specific “internal” or “system” noise is often used to introduce an element of variability in models such as the Computer Model of Object Substitution (CMOS); (Di Lollo et al., 2000). It has never been used as a masking agent (either forward, simultaneous, or backward) in any form of masking (metacontrast, pattern, camouflage, conceptual, etc.) in the vast masking literature. Masking by non-specific noise is certainly not listed in Breitmeyer’s (1984) definitive treatise on masking (Breitmeyer and Öğmen, 2006). More important, it is not mentioned explicitly in Põder’s (2013) Attentional Gating Theory (AGT). To be sure, the claim that internal noise may be a determinant of OSM could be a bold, imaginative step, as long as strong logical and empirical documentation were provided to justify it. As it is, such a claim is *ad hoc* and not part of the AGT as stated in Põder (2013).

### PHENOMENOLOGICAL EVIDENCE

On Põder’s (2013) assumption that the internal representation of the target is degraded because of reduced S/N ratio, one could reasonably expect some distortive effects of the noise to be evidenced in the appearance of the target. In fact, what is seen is a blank area demarcated by the four-dot mask. A compelling description has been provided by Neill et al. (2002, p. 683) as follows:

... in our own experiments the general notion of object substitution is consistent with the phenomenal experience of the masked target: not only does the space inside the dots appear blank, but there is a strong subjective impression of the contours of a square connecting the dots. Furthermore, there is a subjective impression of enhanced brightness of the area within the square, very similar to the brightness enhancement that occurs within illusory contours or subjective contours resulting from long-duration inducing elements (Coren, 1972; Kanizsa, 1976; Petry and Meyer, 1987; Purghe and Coren, 1992).

Such a phenomenological appearance is far from that of a degraded target postulated in Põder’s account. On such an account, additional processes need to be invoked to explain why the reduced S/N ratio causes the target to disappear without a trace instead of appearing merely as degraded. Rather, this phenomenology is precisely what is expected on the basis of OSM: at long-durations of the trailing mask, a mismatch arises between the ongoing pattern of activity at the lower level (four dots alone) and the reentrant perceptual hypothesis (target surrounded by four dots). The mismatch causes that perceptual hypothesis to be discarded and replaced by a new hypothesis consisting of four dots demarcating a blank square area, and that’s what is eventually perceived.

### BEHAVIORAL EVIDENCE

Results inconsistent with the claim that the four-dot mask degrades the target by adding noise to its internal representation have been reported by Lleras and Moore (2003). They showed that OSM was fully in evidence even when the four dots were not physically present around the target after its offset. Rather, what was necessary was the presence of the trailing mask in a location next to the target, under conditions of apparent motion that supported the perception of the target morphing into the mask. Increased noise at the target location can hardly be regarded as a critical determinant of OSM in Lleras and Moore’s study, simply because the target was unobscured by the trailing four-dot mask. Further evidence that OSM occurs when the mask is presented in a location other than that of the target has been reported by Jiang and Chun (2001) and by Guest et al. (2011).

Põder’s assumption that the four dots add noise to the target is also questioned by the results of Bouvier and Treisman (2010) who found that a target’s low-level features can be detected accurately even when OSM prevents identification of the target’s configuration. If, as Põder asserts, a critical factor in OSM were the increased visual noise at the target’s location, what needs to be asked is why the noise spared the target’s low-level features but not its configuration. The likely answer is that OSM interferes with reentrant signaling, leaving the low-level features in the feed-forward sweep largely intact. Evidence consistent with the findings of Bouvier and Treisman has been reported by Guest et al. (2011), and by Binsted et al. (2007) who found that OSM occurs after the physical features of the target have been processed.

More behavioral evidence inconsistent with Põder’s claim that the principal role of the mask is to add noise to the target’s representation has been reported by Jannati et al. (2013). In Experiment 1 of that study, the mask was a solid ring surrounding the target. In Experiment 3, the mask consisted of four small dots, as seen in **Figure 1**. On Põder’s hypothesis, the sizeable contours of the ring should have generated substantially more noise than the sparse contours of the four dots. The strength of masking, therefore, should have been greater in Experiment 1 than in Experiment 3. In fact, the results revealed the opposite pattern, at least numerically.

Another aspect of Jannati et al.’s (2013) study is inconsistent with a key assumption in Põder’s account. Namely, that the amount of noise added to the target is proportional to the mask’s exposure duration. In the study of Jannati et al. (2013) the display sequence began with a brief combined presentation of target and mask, continued with a blank inter-stimulus interval (ISI) of variable duration, and ended with a brief re-presentation of the mask alone. The important point is that, because the duration of the trailing mask was fixed, the amount of noise supposedly added to the target should also have been fixed. This should have given rise to a correspondingly fixed level of OSM. Instead, the results revealed a non-monotonic *U*-shaped function of accuracy over ISI, as predicted in Di Lollo et al.’s (2000) reentrant-processing account.

### ELECTROPHYSIOLOGICAL AND BRAIN-IMAGING EVIDENCE

The electrophysiological and brain-imaging evidence is uniformly supportive of a reentrant-processing account of OSM. To wit, there is broad agreement that OSM interferes with the reentrant sweep while leaving the feed-forward sweep largely unaffected.

Especially relevant to a comparison of reentrant and feed-forward accounts of OSM are two ERP experiments by Woodman and Luck (2003). Experiment 1 employed a search display in which the target was singled out by four dots that either co-terminated with the target or remained on the screen alone for 600 ms after target offset. Two results are directly relevant to the present purpose. First, accuracy of target identification was impaired when the offset of the four-dot mask was delayed (a conventional OSM effect). Second, the target-elicited N2pc (an ERP component said to index target localization, as distinct from target consolidation) was the same in the delayed as in the co-termination conditions. Namely, unlike identification accuracy, the N2pc was unaffected by OSM. This strongly suggested that OSM interfered with later processes of target consolidation, while leaving earlier processes of target localization essentially unaffected. As pointedly noted by Woodman and Luck (2003, p. 608): “The finding of lateralized response to the target (i.e., the N2pc) indicates that on both trial types, the brain was able to determine which side of the array contained the target, which implies that the target was detected by the visual system even though the observers could not accurately report it.”

Põder’s noise-based hypothesis was further disconfirmed in Woodman and Luck’s (2003) Experiment 2 in which the four-dot mask always co-terminated with the target. The critical manipulation was whether or not the target was embedded in visual noise. An important procedural detail was that the strength of the noise was adjusted so that it produced the same degree of behavioral impairment as the delayed-offset mask in Experiment 1.

The results were unambiguous: the N2pc was fully in evidence when the target was unencumbered by visual noise, but was totally absent when the target was embedded in noise. This finding rules out the option that in the delayed-mask-offset condition in Experiment 1 target identification was impaired by visual noise. Had visual noise caused that impairment, it should also have eliminated the N2pc, as it did in Experiment 2. Rather, this pattern of results is consistent with the idea that target identification in Experiment 1 was impaired because the extended four-dot mask interfered with the reentrant signaling. From a reentrant perspective, no suitable perceptual hypotheses could be generated in Experiment 2 when the target was embedded in noise. Whatever perceptual hypotheses were generated consisted largely of visual noise, and that’s what was eventually perceived.

From a broader perspective, it is fitting to ask whether, in principle, four small dots displayed outside the spatial confines of the target can produce sufficient noise to prevent target identification. Or, for that matter, whether they can introduce any manifest noise at all. Experiment 2 of Woodman and Luck (2003) offers important evidence in this respect. In order to match the impairment produced by the extended mask in Experiment 1, the noise mask in Experiment 2 required 23 dots placed directly on top of the target. This raises a further question regarding Põder’s noise-based account of OSM. What needs to be asked is by what means four small dots that remain on view around the target can produce an amount of noise equivalent to that produced by 23 dots placed directly on the target itself. This equivalence cannot be accepted uncritically as stated: it is in need of empirical verification. Similarly, the validity of the claim that four small dots placed as much as 40 min arc away from the target (Di Lollo et al., 2000) can produce sufficient noise to prevent target identification cannot merely be assumed: it needs to be empirically verified.

The idea that OSM interferes with the reentrant sweep while leaving the feed-forward sweep essentially intact is supported by a number of other ERP studies (e.g., Reiss and Hoffman, 2007; Harris et al., 2013). That idea is also buttressed by a functional magnetic-resonance adaptation study by Carlson et al. (2007). Contrary to the hypothesis of increased visual noise at the target’s location, that study revealed no effect of OSM in early visual areas. In contrast, powerful effects of OSM were in evidence at higher cortical regions. Further brain-imaging evidence supportive of the reentrant account of OSM has been reported in an fMRI study by Weidner et al. (2006).

I hasten to note that the evidence listed in the foregoing is not – nor was it intended to be – exhaustive. Rather, the intent was to cite examples of phenomenological, behavioral, electrophysiological, and brain-imaging evidence inconsistent with Põder’s (2013) claim that a critical factor in OSM is the degradation of the internal representation of the target by visual noise generated by the four-dot mask.

### ASSUMPTION OF THE ROLE OF ATTENTION IN OSM

According to Põder (2013), OSM occurs when attention is deployed to the target’s location and finds a representation degraded by visual noise. What is not specified is the mechanism presumed to be involved in the attentional processing.

Attention has been described as a limited resource (Norman and Bobrow, 1975; Lavie and Tsal, 1994), a filter (Broadbent, 1958), a spotlight (Posner et al., 1980), a zoom lens (Eriksen and St. James, 1986), and a glue (Treisman and Gelade, 1980). A major drawback of these metaphors is that they do not specify what underlying mechanisms mediate the purported function. As pointedly noted by Chun et al. (2011, p. 74): “Attention has become a catch-all term for how the brain controls its own information processing....” So, when Põder (2013) invokes “attention” to explain OSM, one is left wondering just what it is that he means. To be useful, accounts of OSM – or, for that matter, accounts of any other phenomenon – should endeavor to make explicit the mechanisms underlying such a nebulous and ill-defined concept as “attention.” It is time to recognize that the indiscriminate use of attention as an explanatory panacea can be an impediment to communication and understanding.

Come to think of it, the function performed by “attention” in Põder’s account of OSM is a more vague – though in some ways equivalent – incarnation of the function performed by reentry in the OSM account of Di Lollo et al. (2000). In the former account, OSM is said to occur when attention is deployed to the target location and finds an item that has been degraded beyond recognition. In the latter, OSM is said to occur when the reentrant signals arrive on their return and find an item that does not match any of the perceptual hypotheses. From a comparison of Põder’s use of “attention” and Di Lollo et al. (2000) use of “reentry” there appears to be a good deal of commonality in the two accounts of OSM.

## CONCLUDING COMMENTS: OF QUANTITATIVE MODELS

Having reviewed the pertinent empirical evidence, we now turn to the quantitative models of OSM: the CMOS proposed by Di Lollo et al. (2000) and the AGT proposed by Põder (2013). CMOS provides an excellent fit to the empirical data illustrated in **Figure 2**; AGT provides an even better fit.

Not to cut too fine a point, it can be confidently stated that both models are misguided. This is because the data that they purport to model (see **Figure 2**) are now known to be vitiated by a confounding. The reasoning is as follows: OSM is defined as the difference in the level of performance observed when the mask co-terminates with the target *minus* the level of performance observed when the mask continues to be on view after target offset. By that criterion, the functions in **Figure 2** indicate that the magnitude of OSM varies with the size of the search display: OSM is maximal at set size 16, and absent at set size 1.

What vitiates the data in **Figure 2** is a response ceiling imposed by the 100% limit of the response scale. When that response ceiling is removed by making the task more difficult, as was done by Argyropoulos et al. (2013; see also Jannati et al., 2013), the functions turn out to be parallel across set sizes. This means that, although the level of performance varies as a function of set size, the magnitude of OSM does not. The invariance of OSM with set size obviously invalidates both the CMOS and the AGT models. Importantly, however, invariance of OSM across set sizes in no way impugns reentry as the underlying mechanism, witness the experimental evidence adduced in the present article.

## Conflict of Interest Statement

The author declares that the research was conducted in the absence of any commercial or financial relationships that could be construed as a potential conflict of interest.

## Footnotes

1 I thank an anonymous reviewer for suggesting the possibility that OSM may arise from internal noise.
